# Regulation of insulin secretion by the post-translational modifications

**DOI:** 10.3389/fcell.2023.1217189

**Published:** 2023-08-04

**Authors:** Chunhua Yang, Mengna Wei, Yanpu Zhao, Zhanyi Yang, Mengyao Song, Jia Mi, Xiaoyong Yang, Geng Tian

**Affiliations:** ^1^ Shandong Technology Innovation Center of Molecular Targeting and Intelligent Diagnosis and Treatment, Binzhou Medical University, Yantai, Shandong, China; ^2^ Yale Center for Molecular and Systems Metabolism, Department of Comparative Medicine, Department of Cellular and Molecular Physiology, Yale University School of Medicine, New Haven, CT, United States

**Keywords:** PTMs, phosphorylation, acetylation, ubiquitination, sumoylation, O-GlcNAcylation, palmitoylation, insulin secretion

## Abstract

Post-translational modification (PTM) has a significant impact on cellular signaling and function regulation. In pancreatic β cells, PTMs are involved in insulin secretion, cell development, and viability. The dysregulation of PTM in β cells is clinically associated with the development of diabetes mellitus. Here, we summarized current findings on major PTMs occurring in β cells and their roles in insulin secretion. Our work provides comprehensive insight into understanding the mechanisms of insulin secretion and potential therapeutic targets for diabetes from the perspective of protein PTMs.

## 1 Introduction

Insulin is an anabolic hormone released from pancreatic islet β cells with the distinct capacity to maintain blood glucose homeostasis (Cabrera et al., 2006; Da Silva Xavier, 2018). The secretion of insulin in response to glucose and other nutrients (such as amino acids and free fatty acids) is a complex process involving the coordination of multiple signaling pathways (Nolan et al., 2006; Henquin, 2011). Impairment of this process is directly associated with the development of diabetes mellitus (Schwartz et al., 2013). Thus, the insulin secretion process is considered as a promising target for the treatment of diabetes mellitus (DeFronzo et al., 2014). But the detail of insulin secretion regulation is still an unanswered question.

Post-translational modification (PTM) is the covalent modification with addition or removal of chemical groups on proteins (Walsh et al., 2005). It is closely associated with almost all physiological and pathological processes by regulating protein localization, degradation, and functions (Walsh and Jefferis, 2006; Khan et al., 2016; Morales-Tarre et al., 2021; Zhu and Hart, 2021). Accumulating evidence suggests that PTMs are extensively involved in the insulin secretion process, Currently, at least eight types of PTMs are known associated with insulin secretion. For example, phosphorylation is required for signaling cascades mediating insulin secretion (Campbell and Newgard, 2021). SUMOylation and palmitoylation have been reported to regulate insulin secretion at multiple stages (Davey et al., 2019; Chamberlain et al., 2021). Acetylation, ubiquitination and O-GlcNAcylation are involved in insulin gene transcription (Mounier and Posner, 2006; Ozcan et al., 2010; Sampley and Ozcan, 2012). Even some understudied PTMs such as citrullination and deamidation are recently reported to be linked with insulin secretion. These indicate that protein PTM plays a critical role in the regulation of insulin secretion in β cells. However, a systematic review of current findings of PTM in insulin secretion is still missing. Here, we review the current understanding of the functional roles of these PTMs in the insulin secretion process.

## 2 Insulin secretion

**FIGURE 1:**
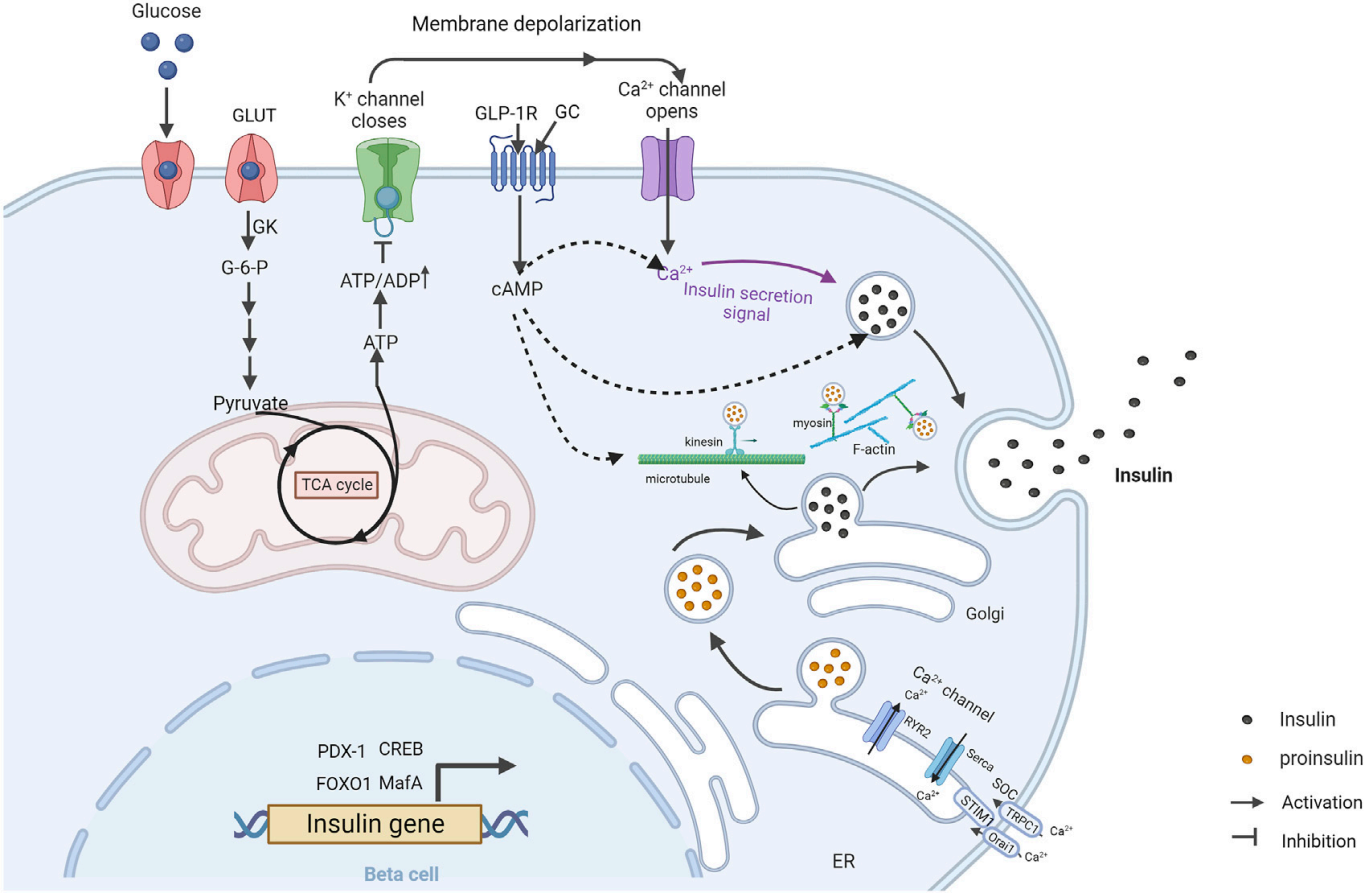
Overview of glucose-stimulated insulin secretion in β cells (Created with BioRender.com). GSIS is mediated by the triggering pathway (solid arrows) and amplification pathways (dashed arrows). At high blood glucose levels, glucose is taken up and converted to glucose-6-phosphate through GK. Glucose-6-phosphate is metabolized by glycolysis and subsequently enters the tricarboxylic acid cycle, triggering an increase in intracellular ATP. Increasing ATP/ADP ratio leads to the closure of the K_ATP_ channel and the opening of Ca^2+^ channels, allowing calcium influx and triggering insulin granules exocytosis. Calcium depletion from ER causes STIM1 to accumulate at ER-plasma membrane junctions where it gates Orai1 and TRPC1, leading to calcium influx and subsequent insulin granules exocytosis. GLP-1 stimulates insulin secretion through cAMP signal pathway. Insulin-vesicles are transported by motor proteins along cytoskeletal biopolymers. Insulin gene transcription is regulated by multiple transcription factors. GC, glucagon; GK, glucokinase; ER, endoplasmic reticulum.

The main physiological stimulus for insulin secretion is blood glucose. Activation of insulin secretion by elevated glucose concentration is called glucose-stimulated insulin secretion (GSIS) (Figure 1). It includes two tandem pathways, the triggering pathway and the amplifying pathway. In the triggering pathway, glucose is transported into islet β cells via glucose transporters (GLUTs) and converted to glucose-6-phosphate (G-6-P) by glucokinase (GK). G-6-P further enters the tricarboxylic acid (TCA) cycle through glycolysis, leading to the production of adenosine triphosphate (ATP). Increased ATP/adenosine diphosphate (ADP) ratio in the cytoplasm causes the closure of the K_ATP_ channel (composed of SUR1 subunits and Kir6.2 subunits) in the cell membrane (Seino, 1999). This leads to the generation of electro-voltage between the inside and outside of the cell membrane, opening voltage-dependent Ca^2+^ channels (VDCCs) and inducing calcium influx (Lim et al., 2009). Subsequently, calcium influx is sensed by multiple calcium-binding proteins and triggers insulin granules exocytosis. The amplifying pathway increases the sensitivity of insulin secretion to the induced calcium influx, which is independent of the K_ATP_ channel (Kalwat and Cobb, 2017). Therefore, the amplifying pathway is also referred to the K_ATP_-independent pathway. However, the exact mechanisms of action of the amplifying pathway are not fully clear (Kalwat and Cobb, 2017). Store-operated calcium channels (SOCs) are believed as a critical compartment of the amplifying pathway. SOCs can be activated by Ca^2+^ depletion from the endoplasmic reticulum (ER) (Prakriya and Lewis, 2015; Lopez et al., 2020). Upon the depletion of ER Ca^2+^ store, stromal interaction molecule 1 (STIM1) aggregates and translocates to the plasma membrane, where it interacts directly with Calcium Release-Activated Calcium Modulator 1 (Orai1) and transient receptor potential canonical channel-1 (TRPC1). This interaction opens SOCs and initiates Ca^2+^ influx, which induces insulin secretion (Tian et al., 2012b). Another critical compartment in the amplifying pathway is the second messenger cyclic adenosine monophosphate (cAMP) (Tian et al., 2015). The cAMP signaling mediates insulin secretion induced by glucose, free fatty acid (FFA) and Glucagon and glucagon-like peptide-1 (GLP-1) (Dyachok et al., 2008; Tian et al., 2011; Tian et al., 2012a).

Insulin secretion in response to glucose stimulation occurs in a biphasic manner composed of a transient first phase and followed a prolonged second phase. Released insulin granules in the first-phase are mainly from a “readily releasable pool” (RRP) in the vicinity of the plasma membrane. Released insulin granules in the second phase are mainly recruited from intracellular storage pools. Actin filaments and microtubules are two main transport routes for insulin granules in β cells (Thurmond et al., 2003; Varadi et al., 2003; Varadi et al., 2005; Cui et al., 2011). Actin filaments mainly contribute to the short-range movement near the plasma membrane, whereas microtubules contribute to the long-range movements (Omar-Hmeadi and Idevall-Hagren, 2021). The remodeling of F-actin cytoskeleton and microtubule network is critical for GSIS in β cells (Thurmond et al., 2003; Bracey et al., 2020; Ho et al., 2020; Wang et al., 2020). Moreover, the formation of SNARE complex which is composed of t-SNARE proteins (SNAP-25 and syntaxin) in the plasma membrane and v-SNARE protein (VAMP) on the insulin granules membrane, is also important for insulin exocytosis.

## 3 Regulatory effect of PTMs on insulin secretion

**FIGURE 2:**
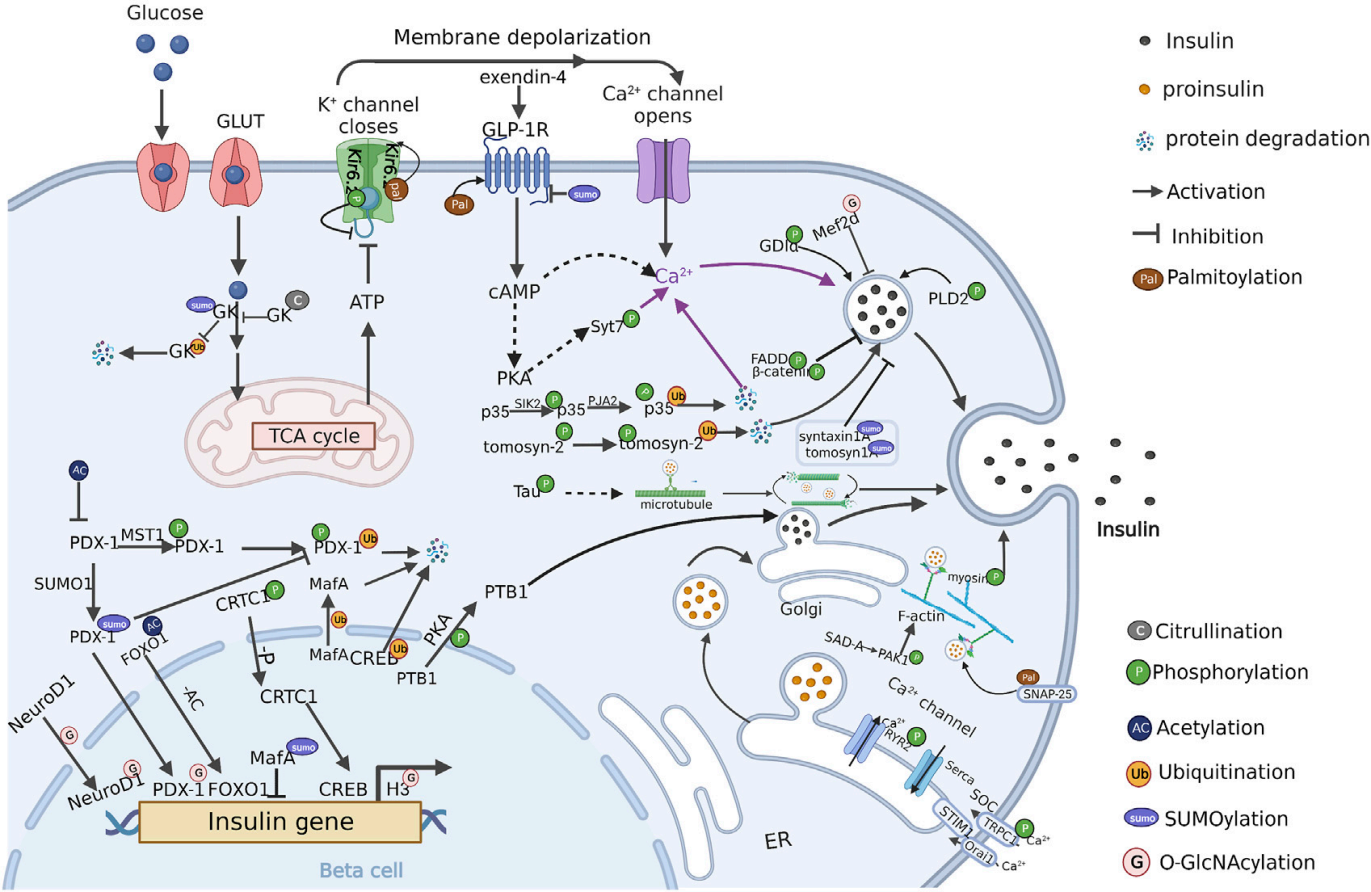
PTM regulation of insulin secretion in β cells (Created with BioRender.com). PTMs are ubiquitously engaged in the various stages of insulin secretion, including signal regulation, insulin gene transcription, insulin-vesicles transportation. Multiple PTMs synergistically regulate insulin secretion in pancreatic β cells. The crosstalk between these PTMs and the modified protein substrates currently known is present in the figure. P: Phosphorylation, AC: Acetylation, Ub: Ubiquitination, SUMO: SUMOylation, G: O-GlcNAcylation, Pal: Palmitoylation, C: Citrullination.

Various PTMs related to insulin secretion have been reported. In the following sections, we will outline currently known PTMs and related substrates in β cells (Table 1), and discuss the roles of individual PTMs in the regulation of insulin secretion (Figure 2).

**TABLE 1:** Summary of PTM regulation in insulin secretion.

Protein name	Protein description	Modification type	Modification function	
			Regulatory direction on insulin secretion	Details
CRTC1	Transcription factor; Regulate insulin gene transcription	Phosphorylation	negative	CRTC is phosphorylated and resides in the cytoplasm
FOXO1		Acetylation	negative	FOXO1 is acetylated and resides in the cytoplasm
CREB		Ubiquitination	negative	Ubiquitination of CREB induces its degradation
PDX-1		Ubiquitination	negative	Ubiquitination of PDX-1 induces its degradation
		SUMOylation	positive	SUMOylation protects PDX-1 from proteasomal degradation
		O-GlcNAcylation	positive	O-GlcNAcylation increase DNA binding activity of PDX-1
MafA		SUMOylation	negative	SUMOylation of MafA results in reduced transcriptional activity
		Ubiquitination	negative	Ubiquitination of MafA induces its degradation
NeuroD1		O-GlcNAcylation	positive	NeuroD1 is O-GlcNAcylated and transfers into the nucleus
histone H3	Core component of nucleosome; Regulate insulin gene transcription	Acetylation	positive	Acetylation increase histone H3 transcriptional activation
		O-GlcNAcylation	positive	O-GlcNAcylation elevates histone H3 transcriptional activation
GK	Glucokinase; Convert glucose to G-6-P, promote TCA cycle and ATP production	Ubiquitination	negative	Ubiquitination of GK induces its degradation
		SUMOylation	positive	SUMOylation protects GK from ubiquitination and degradation
		Citrullination	negative	Citrullination reduces substrate binding affinity of GK
Kir6.2	Component of K_ATP_ channel; Close K_ATP_ channel and induce electro-voltage generation	Phosphorylation	positive	Phosphorylation of Kir6.2 induces inhibition of the K_ATP_ channel conductance
		Palmitoylation	negative	Palmitoylated Kir6.2 increases the open state of K_ATP_
β_2a_	An important auxiliary subunit of VDCCs; Open VDCCs and induce calcium influx	Palmitoylation	positive	Palmitoylation increases the plasma membrane trafficking and location of β_2a_
CDK5R1 (p35)	A cell cycle-dependent protein kinase; Phosphorylates α_1C_ subunit of L-VDCC and inhibits L-VDCC activity	Phosphorylation	positive	Phosphorylation of CDK5R1 triggers its ubiquitination and degradation
Syt7	Cytoplasmic Ca^2+^ sensor; Trigger Ca^2+^-dependent insulin secretion	Phosphorylation	positive	Phosphorylation of Syt7 enhances GLP-1–dependent insulin secretion
RyR2	Component of ER intracellular calcium channels; Mediates the release of Ca2+ from ER into the cytoplasm	Phosphorylates	positive	Phosphorylation of RyR2 promotes Ca2+-dependent insulin secretion
TRPC1	Component of SOCs; Cooperate with Orai1 and STIM1, induce SOCE	Phosphorylation	positive	Phosphorylation of TRPC1 enhances insulin secretion
tau	Microtubule-associated protein; Mediates insulin vesicles movement	Phosphorylation	positive	Tau is phosphorylated and dissociates from microtubules, which promotes microtubules turning over
Myosin	Actin-based molecular motor; Regulate insulin granules trafficking	Phosphorylation	positive	Phosphorylation of MHC and MLC increases insulin secretory granules translocation
β-catenin	A cell-cell adhesion protein; Regulate insulin vesicles trafficking	Phosphorylation	positive	Phosphorylation of β-catenin promotes the rearrangement of the actin cytoskeleton
syntaxin 1A	Part of the SNARE; Participate in the insulin exocytosis	SUMOylation	negative	SUMOylation enhances the binding between syntaxin 1A and tomosyn-1A
tomosyn-1A	Syntaxin 1A binding proteins; Bind with syntaxin 1A to inhibit SNARE complex formation	SUMOylation	negative	
tomosyn-2		Phosphorylation/Ubiquitination	positive	Phosphorylation of tomosyn-2 leads to its ubiquitination and degradation
SNAP-25	A component of SNARE complex; Participate in insulin exocytosis	Palmitoylation	positive	Palmitoylation of SNAP-25 increase its membrane localization
Scamp1	A recycling carrier to the cell surface in post-Golgi recycling pathways; Involved in insulin secretion	Palmitoylation	positive	Palmitoylation of Scamp1 prolonged vesicular kiss-and-run or cavicapture events
PAK1	A protein kinase; Regulate insulin exocytosis as an effector of Rho GTPases	Phosphorylation	positive	Phosphorylation of PAK1 stimulates cytoskeletal remodeling required for insulin exocytosis
PTB1	A mRNA binding protein; Regulate secretory granule proteins level	Phosphorylation	positive	Phosphorylation enhances the binding activity of PTB1 to the 3′UTR of mRNAs encoding secretory granule proteins
GLP-1R	GLP-1 reporter; Regulating insulin secretion in response to GLP-1	SUMOylation	negative	SUMOylation attenuates cell surface trafficking of GLP-1R
		Palmitoylation	positive	Palmitoylation induces GLP-1R clustering, nanodomain signaling, and internalization
GDIα	Locks Rho GTPases in an inactive GDP-bound form and inhibits GSIS	Phosphorylation	positive	GDIα is phosphorylated and dissociates from Rho GTPases, leading to the onset of GSIS
PLD2	A phospholipase; Hydrolyzes phosphatidyl choline to generate phosphatidic acid, a mediator of insulin exocytosis	Phosphorylation	positive	Phosphorylation of PLD2 activates EGF-dependent insulin secretion
FADD	An apoptotic adaptor molecule	Phosphorylation	negative	Phosphorylation of FADD induces impaired GSIS
IAPP	Islet amyloid polypeptide; Co-expresses and secretes with insulin; Induces toxicity toward β cells and inhibits insulin secretion	Deamidation	negative	Deamidation modulates IAPP amyloid formation and fibril morphology, inducing its cytotoxicity

### 3.1 Phosphorylation

Phosphorylation is a ubiquitous PTM regulated by protein kinases and phosphatases that transfer and remove phosphate groups from ATP or GTP to amino acid residues (Ser, Tyr, Thr) of proteins, respectively. It is widely involved in signal transduction pathways and associated with various physiological and pathological processes by regulating cell proliferation, development, differentiation, apoptosis, and other cell processes (Humphrey et al., 2015). Phosphorylation mediated signaling cascades play an important role in maintaining glucose homeostasis (Roder et al., 2016). The insulin/insulin receptor and their downstream proteins, such as PI3K/AKT and ERK, regulate various metabolic pathways including improving glucose tolerance and protecting against insulin resistance (Dall'Agnese et al., 2022). In the insulin secretion process, phosphorylation is wildly existing in the insulin biosynthesis and signaling cascade of insulin exocytosis (Sacco et al., 2016).

Transcription factors are required in insulin gene transcription, and regulated by phosphorylation (Knoch et al., 2006; Ch’ng et al., 2012; Malm et al., 2016). cAMP-Regulated Transcriptional Co-activator-1 (CRTC1) is a cAMP-responsive element binding protein (CREB)-mediated transcription regulator. Under basal conditions, CRTC is phosphorylated by Salt Inducible Kinase (SIK) and binds to 14-3-3 proteins, sequestering in the cytoplasm (Ch’ng et al., 2012; Malm et al., 2016). Dephosphorylation of CRTC by activating CRTC phosphatase or inhibiting CRTC kinases triggers its dissociation from 14-3-3 and translocation into the nucleus, where it activates CREB-mediated transcription of *Ins1* gene (Oetjen et al., 1994; Altarejos et al., 2008; Altarejos and Montminy, 2011). It has been reported that this process is involved in GLP-1-stimulated insulin secretion (Shin et al., 2014). Cytosolic polypyrimidine tract-binding protein 1 (PTB1) binds and stabilizes mRNA encoding secretory granules (SGs) (Knoch et al., 2004). PKA-dependent PTB1 phosphorylation is promoted by elevated cAMP and in turn promotes SG expression and insulin secretion (Knoch et al., 2006).

In β cells, Ca^2+^ is considered as triggering signal of insulin exocytosis. Phosphorylation is involved in the depolarization of membrane potential and calcium influx. Kir6.2, the K_ATP_ channel component, can be phosphorylated on Ser385 by AMPK. Phosphorylated Kir6.2 restrains K_ATP_ channel activity and leads to depolarization of membrane potential. This depolarization results in the opening of VDCCs, leading to elevated intracellular calcium levels and ultimately induced insulin secretion (Chang et al., 2009). Synaptotagmin-7 (Syt7) is a major cytoplasmic Ca^2+^ sensor for exocytosis by triggering secretory granule fusion and insulin secretion (Gao et al., 2000; Gauthier et al., 2008). It is reported that GLP-1 stimulates PKA-dependent phosphorylation of Syt7 at Ser103. Such modification enhances Ca^2+^-triggered exocytosis, whereas dephosphorylation of Syt7 disrupts GLP-1 potentiation of insulin secretion (Wu et al., 2015). Besides cytoplasmic Ca^2+^ balance, calcium homeostasis in the ER also has a pivotal role in insulin secretion (Sabourin et al., 2015; Li et al., 2021). Ryanodine receptors (RyRs) are vital components of ER intracellular calcium channels which mediate Ca^2+^ release from ER into the cytoplasm (Doser et al., 2020). RyR2 is one of the RyRs isoforms that is expressed in β cells (Johnson et al., 2004; Dror et al., 2008; Takasawa et al., 2010). It has been shown that sufficient phosphorylation of RyR2 and subsequent Ca^2+^ release are essential steps in GSIS (Dixit et al., 2013; Llanos et al., 2015). However, hyperphosphorylation of RyR2 induced by CaMKII leads to glucose intolerance, impaired GSIS and lowered [(Ca^2+^)]_cyt_ transients. This is due to the increased basal RyR2-mediated Ca^2+^ leak and basal hyperinsulinemia (Dixit et al., 2013). Moreover, components of SOCs can be phosphorylated. TRPC1, as a component of SOCs, can be phosphorylated by protein kinase C (PKC). Inhibition of PKC activity reduces TRPC1 phosphorylation and decreases insulin secretion rapidly, which could be restored by the TRPC1 activator (Xu et al., 2019).

Insulin granules transport and release also can be regulated by protein phosphorylation (Ho et al., 2020). Myosin is a motor protein that is responsible for insulin granules transportation dependent on F-actin (Varadi et al., 2005; Sweeney and Holzbaur, 2018; Omar-Hmeadi and Idevall-Hagren, 2021). It is composed of heavy chains (MHC) and two types of light chains (MLC) (Penn et al., 1982; Niki et al., 1993). Both MHC and MLC can be phosphorylated by multiple protein kinase including Myosin light-chain kinase (MLCK), and promote insulin secretion (Tan et al., 1992; Iida et al., 1997; Wilson et al., 1999). Tau is a microtubule-associated protein (MAP) involved with insulin secretion and glycemic control (Maj et al., 2016; Wijesekara et al., 2018). High glucose induces hyper-phosphorylation of tau by multiple kinases including GSK3, PKA, PKC, and CDK5, which enhances microtubule turnover to acutely induces GSIS (Ho et al., 2020). β-catenin, as a cell-cell adhesion protein, increases GSIS through promoting the rearrangement of actin cytoskeleton (Sorrenson et al., 2016). It can be phosphorylated at Ser552 by p21-activated protein kinase-1 (PAK1) in response to glucose and GLP-1 stimulation. A mutation of Ser552 to Ala on β-catenin attenuates GSIS, suggesting a critical role for β-catenin Ser552 phosphorylation in insulin secretion (Sorrenson et al., 2021). Tomosyn is a negative regulator of cellular exocytosis and can hinder insulin secretion (Zhang et al., 2006; Bhatnagar et al., 2011). In response to high glucose, enhanced tomosyn-2 phosphorylation targets tomosyn-2 for Hrd-1-mediated ubiquitination and degradation, causing increased insulin secretion (Bhatnagar et al., 2014). Activated Phospholipase D2 (PLD2) can stimulate insulin secretion (Jones et al., 1999). PLD2 phosphorylation at Ser134 via cell cycle protein-dependent kinase 5 (CDK5) plays an influential role in EGF-dependent insulin secretion (Lee et al., 2008). Brain-selective kinase 2 (BRSK2), also known as SAD-A, is a member of the AMPK-related kinase family. It is abundantly expressed in pancreatic islets β cells and acts a key function in insulin secretion (Lizcano et al., 2004). Overexpression of SAD-A significantly enhances GSIS and further potentiates GLP-1’s effect on GSIS in isolated mouse islets (Nie et al., 2013). Phosphorylation of PAK1 at Thr423 via SAD-A triggers the onset of GSIS in islet β cells (Nie et al., 2012). Phosphorylation of GDP-dissociation inhibitors (GDIα) at Ser174 by SAD-A leads to the dissociation of Rho GTPases from GDIα complexes, culminating in insulin exocytosis (Nie et al., 2018).

Phosphorylation also presents an inhibitory role in insulin secretion. Fas-associated death domain protein (FADD) is a classical adaptor in Fas-FasL signaling, which can regulate islet mass and insulin secretion. In the mouse model of FADD-D (S191D), which mimics the constitutive expression of phosphorylated FADD in mice, the area of pancreatic islets is shrunken, and GSIS is impaired. This suggests that FADD phosphorylation negatively regulates islet development and insulin secretion (Yao et al., 2015).

### 3.2 Acetylation

Acetylation is a chemical reaction in which an acetyl group is added to a compound in place of a hydrogen atom, which can be regulated by histone acetyltransferases (HATs) and histone deacetylases (HDACs) (Gao et al., 2017). Acetylation not only regulates histones, transcription factors, and epigenetic regulators, but also regulates many enzymes in metabolic pathways such as glycolysis, gluconeogenesis, tricarboxylic acid cycle, and fatty acid oxidation (Guan and Xiong, 2011).

Protein acetylation is tightly linked to insulin secretion and functional regulation of pancreatic β cells (Zhang et al., 2019). As a class of highly conserved deacetylases, Sirtuins (SIRTs) significantly contribute to insulin secretion. Among the SIRT family, SIRT1 is located in the nucleus, and ameliorates hyperglycemia by promoting insulin secretion and β cell expansion (Luu et al., 2013). SIRT2 is the only predominantly cytoplasmic isoform but is also found in the nucleus and mitochondria (Vaquero et al., 2006; Liu et al., 2017). SIRT2 knockout rats exhibit impaired glucose tolerance and decreased GSIS (Moynihan et al., 2005). SIRT3 and SIRT4 are mainly localized in the mitochondria. Overexpression of SIRT3 inhibits acetylation and degradation of trifunctional enzyme subunit alpha (ECHA) that participated in fatty acid β-oxidation, resulting in increased β-oxidation of fatty acid and reduced oxidation of glucose in β cells. SIRT3 knockout mice show increased insulin secretion upon glucose stimulation (Zhang et al., 2019). SIRT4 regulates amino acid catabolism and insulin secretion, maintaining glucose homeostasis during aging (Haigis et al., 2006; Anderson et al., 2017). SIRT6 is mainly localized in the nucleus. Pancreatic β cell-specific SIRT6 knockout mice show significantly reduced GSIS (Xiong et al., 2016).

Most known acetylated proteins contributing to insulin secretion are transcription factors. Pancreatic and duodenal homeobox-1 (PDX-1) and FOXO1 are two transcription factors associated with insulin gene transcription (Sussel et al., 1998; Berneman-Zeitouni et al., 2014). The acetylated form of FOXO1 resides in the cytoplasm while the deacetylated form is mainly in the nucleus. Deacetylation of FOXO1 enhances its transcriptional activity and plays an essential role in insulin signaling (Accili and Arden, 2004). Moreover, insulin secretion is enhanced in deacetylated FOXO1 (6 KR) knock-in mice (Kim-Muller et al., 2016). lncRNA MALAT1 impairs insulin secretion by reducing histone H3 acetylation at the *Pdx-1* promoter and subsequently inhibiting *Pdx-1* expression (Ding et al., 2020).

### 3.3 Ubiquitination

Ubiquitination is regulated by a three-step cascade: ubiquitin activation by E1 enzymes, conjugation by E2 enzymes, and ligation by E3 ligases (Komander and Rape, 2012). The Ubiquitin-proteasome system (UPS) is the main pathway for intracellular protein degradation. It plays a crucial role in the regulation of glucose homeostasis, insulin secretion, and the pathogenesis of diabetes mellitus (Price et al., 1996; Kawaguchi et al., 2006; Song et al., 2013; Uruno et al., 2013; Al-Khalili et al., 2014; Yang et al., 2016; Balaji et al., 2018; Yamada et al., 2018).

A group of critical proteins involved in insulin secretion is regulated by ubiquitination-mediated degradation. GK degradation by ubiquitination inhibits insulin secretion by decreasing G-6-P production (Cho et al., 2020). The proteasome inhibitor lactacystin has been shown to enhance GSIS in a 2-h short-term treatment (Lopez-Avalos et al., 2006). The v-maf musculoaponeurotic fibrosarcoma oncogene homolog A (MafA) is a key transcription factor required for β cells formation and function (Zhang et al., 2005; Andrali et al., 2008; Kaneto and Matsuoka, 2015). MafA deletion in mice causes glucose intolerance and induces diabetes. The E3 ubiquitin ligase Hrd-1 targets MafA for ubiquitination and degradation in β cells, which results in cytoplasmic accumulation of MafA. Such accumulation impairs its function in the nucleus, causing reduced insulin secretion (Wu et al., 2020). CREB is a transcription factor that is essential for glucose homeostasis and β cell survival (Jambal et al., 2003; Jhala et al., 2003; Sarkar et al., 2007). Chronic hyperglycemia increases CREB ubiquitination and decreases protein expression which ultimately inhibits insulin secretion (Costes et al., 2009). Somatostatin receptor subtype 5 (SSTR5) inhibits PDX-1 expression by downregulating *Pdx-1* transcription and enhancing PDX-1 ubiquitination at post-translational level, thus reducing insulin secretion (Zhou et al., 2012). Phosphorylation of PDX-1 at Thr11 by Macrophage Stimulating 1 (MST1) in β cells leads to PDX-1 ubiquitination and degradation, resulting in impaired insulin secretion (Ardestani et al., 2014). A previous report showed that silencing CDK5 activator CDK5R1 (also known as p35) enhances insulin secretion in MIN6 cells in high glucose culture (Wei et al., 2005). Subsequent research showed that p35 phosphorylation via Salt-inducible kinase 2 (SIK2) induces p35 ubiquitylation. The modification is mediated by the E3 ubiquitin ligase PJA2, which leads to the activation of calcium entry and insulin secretion (Sakamaki et al., 2014).

### 3.4 SUMOylation

SUMOylation is a post-translational modification of small SUMO proteins that is catalyzed by activating enzyme (E1), conjugating enzyme (E2), and ligase (E3), and reversed by specific proteases such as Sentrin-specific SUMO proteases (SENPs) (Li et al., 2005; Flotho and Melchior, 2013). It plays an important role in modulating protein activity, protein-protein interactions, and subcellular localization (Shao and Cobb, 2009; Gareau and Lima, 2010). It has been reported that the homeostasis of SUMOylation plays an essential role in maintaining β cell function (Dai et al., 2011; Rajan et al., 2012; Vergari et al., 2012; Davey et al., 2019).

E2 SUMO-conjugating enzyme (UBC9) is the only conjugating enzyme essential for the SUMO system. Mice lacking UBC9 in β cells exhibit decreased insulin content and loss of β cell mass. In contrast, overexpression of UBC9 in β cells leads to an increased antioxidant ability but impaired insulin secretion (He et al., 2018). Protein deSUMOylation by SENPs regulates the conjugation/deconjugation balance of target proteins. Studies on islet-specific SENP1 deletion in mice further demonstrated that the knockdown of SENP1 reduces Ca^2+^-triggered β cell exocytosis. Conversely, overexpression of SENP1 augments β cell exocytosis (Vergari et al., 2012). It suggests a key role for SUMOylation/deSUMOylation balance in GSIS.

SUMOylation regulates insulin secretion at multiple stages. In the insulin gene transcription process, SUMOylation reduced transcriptional activity of MafA toward the insulin gene promoter in low glucose (2 mm) or exposure to hydrogen peroxide (Shao and Cobb, 2009). However, SUMOylation protects PDX-1 from proteasomal degradation and promotes its entry into the nucleus, where it activates insulin gene transcription (Kishi et al., 2003). SUMOylation also participates in triggering insulin secretion pathway. Overexpression of SUMO-1 in β cells increases the stability and activity of GK to induce the closure of the K_ATP_ channel (Aukrust et al., 2013), meanwhile exert a strong inhibitory action on the K_v_2.1 voltage-dependent K^+^ channel (MacDonald et al., 2001; MacDonald et al., 2002; Dai et al., 2009). The depolarization of cell membrane promotes the activation of calcium channels and subsequent insulin secretion. GLP-1 activates the GLP-1 signaling by the interaction with GLP1 receptor (GLP-1R) in β cells, resulting in a rapid increase in intracellular cAMP that promotes insulin secretion. Overexpression of SUMO-1 attenuates GLP-1R function by preventing GLP-1R oligomerization which is essential for forward trafficking, leading to a significant reduction in insulin secretion (Rajan et al., 2012). SUMOylation has been proposed to act as a ‘brake’ on insulin exocytosis. Some proteins associated with insulin exocytosis have been reported to be SUMOylated such as tomosyn1A and syntaxin 1A. SUMOylation increases the interaction between tomosyn1A and syntaxin 1A, which sequesters syntaxin 1A to inhibits the formation of SNARE complex and subsequent insulin exocytosis (Ferdaoussi et al., 2017; Davey et al., 2019). Moreover, SUMOylation suppresses Syt7-mediated insulin secretion, which is transiently lost upon glucose stimulation and returns within 30–60 min (Dai et al., 2011).

### 3.5 O-GlcNAcylation

O-GlcNAcylation is a unique protein glycosylation that is controlled bidirectionally by two enzymes: the writer O-GlcNAc transferase (OGT) and the eraser O-GlcNAcase (OGA). As a nutrition sensor, O-GlcNAcylation is closely associated with type 2 diabetes and associated complications (Dias and Hart, 2007).

O-GlcNAc homeostasis in β cells plays a notable role in insulin secretion (Sireesh et al., 2014; Humphrey et al., 2015; Wende, 2015; Yang and Qian, 2017; Yoshida et al., 2022). OGT is expressed virtually in all cell types but is particularly high in pancreatic β cells (Hanover et al., 1999; Hart and Akimoto, 2009). β cell-specific OGT deletion leads to reduced insulin secretion, and this effect is more pronounced in high fat diet-fed mice (Lockridge et al., 2020). OGA acts in opposition to OGT to regulate protein O-GlcNAcylation. Research has shown that overexpression of OGA in β cells decreases insulin secretion and impairs glucose tolerance in mice (Soesanto et al., 2011). Paradoxically, OGA deletion in pancreatic β cells also impairs insulin secretion *in vivo* and *in vitro* (Yoshida et al., 2022).

Currently, most identified substrates of O-GlcNAcylation are related to insulin gene transcription. Increased nuclear O-GlcNAcylation increases intracellular insulin levels and reserves GSIS in part by boosting histone H3 transcriptional activation to promote *Ins1* and *Ins2* gene transcription in Min6 cells (Durning et al., 2016). Glucose induced PDX-1 O-GlcNAcylation leads to increased PDX-1 DNA binding activity and insulin secretion in Min6 cells (Gao et al., 2003). Further studies demonstrated that OGT interacts with phosphatidylinositol 3,4,5-trisphosphate (PIP3), which enables OGT to catalyze O-GlcNAcylation of nuclear proteins, including PDX-1 (Yang et al., 2008; Kebede et al., 2012). The localization of NeuroD1, a transcription factor of the insulin gene, is regulated by O-GlcNAcylation (Peterson and Hart, 2016). Under low glucose conditions, NeuroD1 is mainly in the cytosol. However, OGA inhibitor treatment induces NeuroD1 translocation into the nucleus, leading to enhanced insulin expression (Andrali et al., 2007). Another transcription factor, myocyte enhancer factor 2D (Mef2d), has been reported to negatively regulate insulin secretion through O-GlcNAcylation (Yoshida et al., 2022).

### 3.6 Palmitoylation

Palmitoylation, the attachment of fatty acyl chains to cysteine residues, is a reversible process mediated by the opposing activities of acyltransferases and thioesterases (Chamberlain and Shipston, 2015). Palmitoylation is a recently discovered PTM that plays a significant role in the regulation of cellular functions (Blaskovic et al., 2014; Chamberlain and Shipston, 2015). Recent studies have revealed the emerging importance of palmitoylation in insulin secretion and insulin response pathways (Chamberlain et al., 2021; Dong et al., 2023). Acyl-protein thioesterase 1 (APT1) is a depalmitoylation enzyme. The function of APT1 was blocked in chronic hyperglycemia, leading to defective insulin secretion (Chamberlain et al., 2021). APT1 knockdown in islets caused insulin hypersecretion (Dong et al., 2023).

In β-cells, palmitoylation are widely present in ion channels and insulin exocytosis. Kir6.2 is palmitoylated at cysteine-166, which increase the open state of the K_ATP_ channel and lead to inhibitory insulin secretion (Chamberlain et al., 2021). β_2a_, an important auxiliary subunit of VDCCs, is palmitoylated at cysteine-3/4, thus increasing plasma membrane trafficking of β_2a_ and Cav subunits of VDCCs. However, excessive number of palmitoylated Ca_V_β_2a_ leads to Ca^2+^ overload and β cell death (Kazim et al., 2017). Palmitoylation of SNAP-25 in the central region increase its membrane localization, which may help insulin exocytosis (Gonelle-Gispert et al., 2000). Scamp1, which is localized in insulin secretory granules, can be palmitoylated at Cys132. Palmitoylation-defective Scamp1 mutant C132S rescues insulin hypersecretion and nutrient-induced apoptosis in APT1-deficient cells (Dong et al., 2023). GLP-1R is palmitoylated mainly at Cys438 in response to agonists, inducing GLP-1R clustering, nanodomain signaling, and internalization. These result in enhanced GLP-1R signaling and insulin secretion (Buenaventura et al., 2019).

### 3.7 Other PTMs

Besides the PTM types described above, a group of less-studied PTM types is also related to insulin secretion. Citrullination is mediated by calcium-dependent peptidyl arginine deiminases (PADs), which catalyze deimination, the conversion of arginine into non-classical amino acid citrulline (Kunieda et al., 2018). A major role for PADs and citrullinated proteins has emerged in type 1 diabetes (Yang et al., 2021). Citrullination alters the enzyme kinetics of GK and suppresses GSIS (Yang et al., 2022).

Deamidation is the conversion of glutamine to glutamic acid by transglutaminase (TGM) enzymes (Callebaut et al., 2022). Deamidated peptides have been reported as autoantigens in type 1 diabetes (Callebaut et al., 2022). Islet amyloid polypeptide (IAPP) accelerates the pathogenesis of type 2 diabetes by exacerbating β cell degeneration and ultimately compromising insulin secretion. Studies have revealed that deamidation can modulate IAPP amyloid formation and fibril morphology, which induces its cytotoxicity (Nguyen et al., 2017).

## 4 Conclusion and perspectives

In this review, we summarized the current progress of PTM regulation in insulin secretion. Eight PTMs and at least twenty-nine modified protein substrates have been reported to be associated with insulin secretion, suggesting that insulin secretion is extensively regulated by PTMs. However, there remains numerous unanswered questions about the role of PTMs in insulin secretion. For example, currently more than 600 PTMs have been identified in eukaryotes (Bradley, 2022). Whether there are more PTMs and more modified substrates present in β cells? What’s the function? Furthermore, it is noticed that some key proteins are regulated by multiple PTMs with consistent or contrary effect. What’s the crosstalk between the different PTMs? How these PTMs synergistically regulate the function of protein? At last, defective or excessive PTMs may induce insulin deficiency, or insulin hypersecretion which may lead to β cells failure, resulting impaired insulin secretion. How the PTMs are dynamically regulated in the physiologic or pathological insulin secretion process? Moreover, several drug candidates for T2DM have been proved to regulate protein PTMs. For example, Glucokinase activators (GKAs), including dorzagliatin, MK-0941 and AZD1656, also promotes SUMOylation of pancreatic glucokinase, (Wu et al., 2023), implying PTM can be the potential drug targets for T2DM. Thus, further in-depth studies of PTMs in insulin secretion are in need.
